# Germline biallelic *Mcm8* variants are associated with early-onset Lynch-like syndrome

**DOI:** 10.1172/jci.insight.140698

**Published:** 2020-09-17

**Authors:** Mariano Golubicki, Laia Bonjoch, José G. Acuña-Ochoa, Marcos Díaz-Gay, Jenifer Muñoz, Miriam Cuatrecasas, Teresa Ocaña, Soledad Iseas, Guillermo Mendez, Daniel Cisterna, Stephanie A. Schubert, Maartje Nielsen, Tom van Wezel, Yael Goldberg, Eli Pikarsky, Juan Robbio, Enrique Roca, Antoni Castells, Francesc Balaguer, Marina Antelo, Sergi Castellví-Bel

**Affiliations:** 1 Oncology Section and; 2 Molecular Biology Laboratory, Hospital of Gastroenterology “Dr. C.B. Udaondo,” Buenos Aires, Argentina.; 3 Gastroenterology Department, Institut d’Investigacions Biomèdiques August Pi i Sunyer (IDIBAPS), Centro de Investigación Biomédica en Red de Enfermedades Hepáticas y Digestivas (CIBEREHD), Hospital Clínic, Universitat de Barcelona, Barcelona, Spain.; 4 Pathology Department, IDIBAPS, CIBEREHD, and Tumor Bank-Biobank, Hospital Clínic, Barcelona, Spain.; 5 Department of Pathology and; 6 Department of Clinical Genetics, Leiden University Medical Center, Leiden, Netherlands.; 7 Recanati Genetics Institute, Rabin Medical Center, Petah Tikva, Israel.; 8 Lautenberg Center for Immunology and Cancer Research, Institute for Medical Research, Israel-Canada, Hebrew University-Hadassah Medical School, Jerusalem, Israel.

**Keywords:** Gastroenterology, Genetics, Colorectal cancer, DNA repair, Genetic diseases

## Abstract

Lynch syndrome is the most common cause of hereditary colorectal cancer (CRC), and it is characterized by DNA mismatch repair (MMR) deficiency. The term Lynch-like syndrome (LLS) is used for patients with MMR-deficient tumors and neither germline mutation in *MLH1*, *MSH2*, *MSH6*, *PMS2,* or *EPCAM* nor *MLH1* somatic methylation. Biallelic somatic inactivation or cryptic germline MMR variants undetected during genetic testing have been proposed to be involved. Sixteen patients with early-onset LLS CRC were selected for germline and tumor whole-exome sequencing. Two potentially pathogenic germline *MCM8* variants were detected in a male patient with LLS with fertility problems. A knockout cellular model for *MCM8* was generated by CRISPR/Cas9 and detected genetic variants were produced by mutagenesis. DNA damage, microsatellite instability, and mutational signatures were monitored. DNA damage was evident for *MCM8^KO^* cells and the analyzed genetic variants. Microsatellite instability and mutational signatures in *MCM8^KO^* cells were compatible with the involvement of *MCM8* in MMR. Replication in an independent familial cancer cohort detected additional carriers. Unexplained MMR-deficient CRC cases, even showing somatic biallelic MMR inactivation, may be caused by underlying germline defects in genes different than MMR genes. We suggest *MCM8* as a gene involved in CRC germline predisposition with a recessive pattern of inheritance.

## Abstract 2

MCM8 may be involved in germline predisposition to colorectal cancer in Lynch-like syndrome cases.

## Graphical abstract

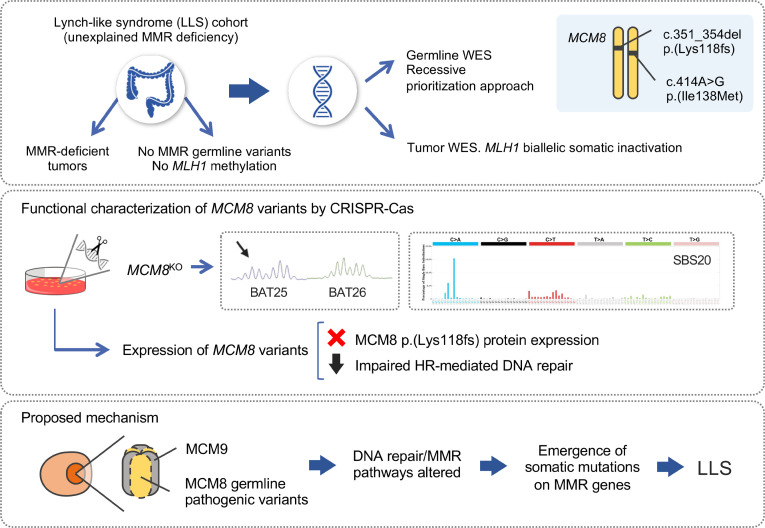

## Introduction

Lynch syndrome (LS) is the most frequent cause of hereditary colorectal cancer (CRC), accounting for 3% of all stages of CRC (1). LS is an autosomal dominant condition caused by germline pathogenic variants in one of the DNA mismatch repair (MMR) system genes or the *EPCAM* gene (2). Although *MSH2* and *MLH1* account for most of the LS-associated CRC cases, *PMS2* and *MSH6* variants are actually more prevalent on a population basis (3). This syndrome has a marked gene-dependent variable penetrance for CRC and endometrial carcinoma (12%–55%) and an increased risk for various other extracolonic tumors (4). Surveillance colonoscopies every 2 years starting at age 25–35 and yearly endometrial screening from age 40 are advised to reduce morbidity and mortality related to cancer (5).

Tumors of patients with LS display microsatellite instability (MSI), caused by the initial germline inactivation and a second somatic hit in the other allele of one of the MMR genes (2). However, the MSI specificity for LS is low because it also occurs in 12%–15% of sporadic CRC cases, usually due to somatic *MLH1* promoter region hypermethylation. Nevertheless, for a MSI-positive CRC, absence of *MLH1* somatic hypermethylation or MMR germline pathogenic variants may be as common as 70% (6). MMR deficiency (MMRd) in tumors could be due to a sporadic “chance” event (evidenced by somatic mutations in *BRAF*, double somatic DNA MMR genes, or *MLH1* promoter hypermethylation) or caused by an underlying “syndromic” predisposition to MMRd tumors by undetected germline defects in either the DNA MMR genes known to be associated with LS or others. The cases in the latter option have been termed “Lynch-like syndrome” (LLS), and management decisions in these patients and their families are complicated because of a suspected but unconfirmed hereditary origin (7).

Therefore, 3 possibilities could explain a MMRd tumor: (a) cryptic/undetected germline mutation in DNA MMR gene (occult/undetected LS), (b) double somatic mutation in DNA MMR gene (sporadic MMRd) in 50%–60% of the cases (8–10), and (c) mutations (germline) in other genes involved in DNA MMR (heritable predisposition). The last 2 hypotheses may overlap in some patients, and it is unclear if this is the case in early-onset LLS cases.

Keeping in mind the last possibility for a MMRd tumor and to further explore it, the aim of the present study was to investigate if patients with early-onset LLS carried potentially pathogenic germline variants in CRC predisposition genes, thereby causing a MMRd tumor phenotype. Accordingly, we performed exome sequencing in the germline and tumor DNA of 16 patients with early-onset LLS CRC and carried out an exhaustive functional evaluation for 2 rare recessive germline variants detected in a candidate gene.

## Results

### Clinical characteristics and germline sequencing results.

We selected 16 patients with nonpolyposis LLS CRC, who were diagnosed before the age of 40. These patients presented tumors with MSI and/or IHC loss of MLH1, MSH2, MSH6 or PMS2, WT *BRAF* V600E and/or negative *MLH1* methylation, and with no germline pathogenic variants in the MMR or *EPCAM* genes.

**Figure 1:**
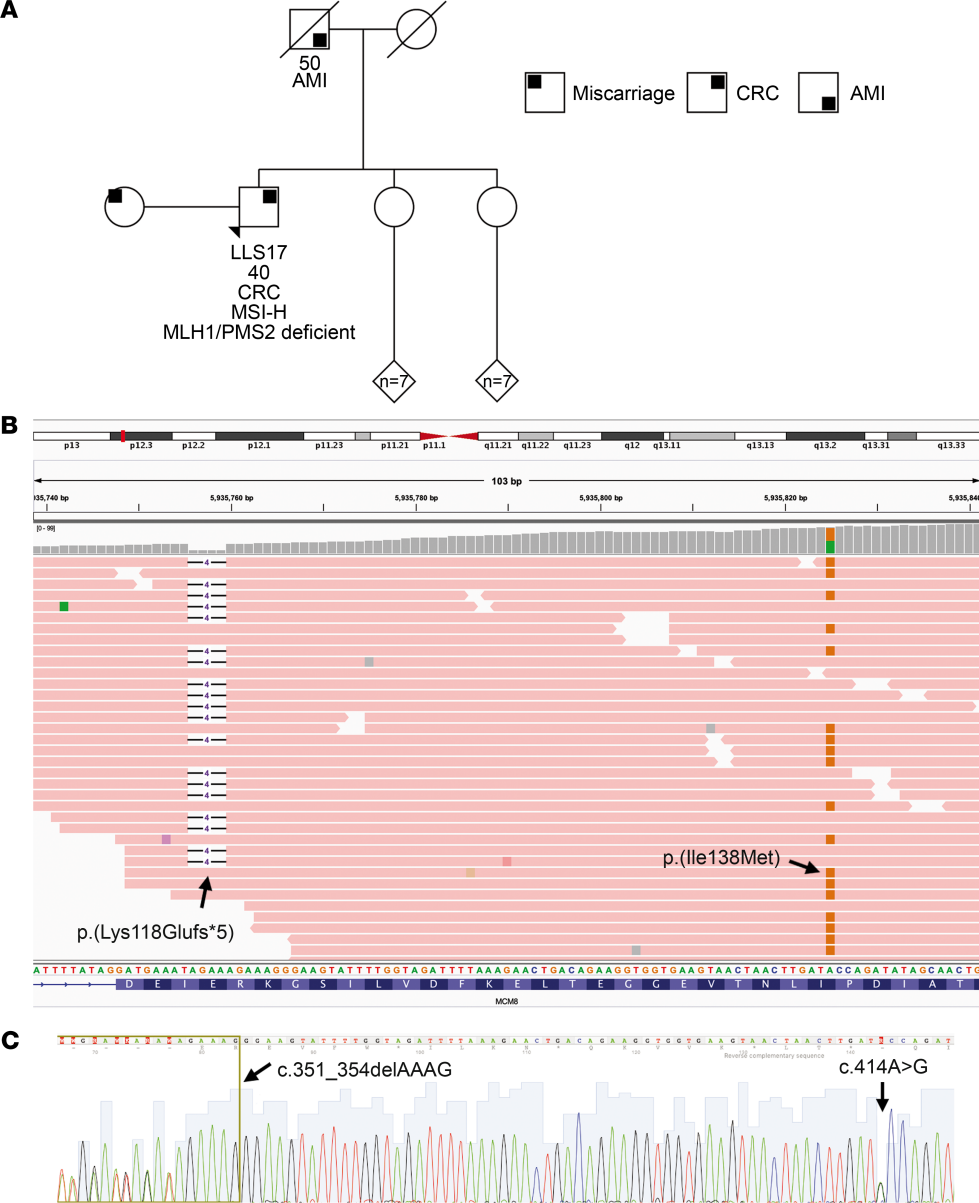
Family tree and germline sequencing results. (**A**) LLS17 family. Black arrow indicates CRC patient carrying the germline MCM8 variants. (**B**) Screenshot showing that the biallelic variants are in trans. The variants are mutually exclusive when a single read crosses both positions. (**C**) Sanger validation of MCM8 germline biallelic variants p.(Lys118Glufs*5) and p.(Ile138Met) (c.351_354delAAAG and c.414A>G). LLS, Lynch-like syndrome; CRC, colorectal cancer; AMI, acute myocardial infarction; MSI-H, microsatellite instability high.

Germline whole-exome sequencing (WES) was performed in all DNA samples. A recessive analysis was prioritized in the analyzed patients because the family pattern was more compatible with an autosomal recessive inheritance. After variant prioritization, germline WES data analysis selecting for variants located on genes with a function compatible with cancer development yielded 2 potentially pathogenic *MCM8* variants in a male patient with early-onset CRC LLS (LLS17, Figure 1A). Patient LLS17 presented an IIIB stage (T4N1M0), left-sided colon, well-differentiated, mucinous adenocarcinoma; was diagnosed at 40 years of age; referred no cancer family history; and reported fertility problems with his spouse, who was suffering an advanced pregnancy miscarriage. The *MCM8* variants identified were p.(Lys118Glufs*5) and p.(Ile138Met). Manual visualization of WES data showed they were in trans and mutually exclusive when a single sequencing read passed through both genomic positions (Figure 1B). Both MCM8 variants were validated by Sanger sequencing (Figure 1C). The p.(Lys118Glufs*5) frameshift variant was predicted pathogenic and very rare in the general population (11/276,744). On the other hand, the missense p.(Ile138Met) variant was also in silico predicted as deleterious and destabilizing for the protein as well as moderately rare in controls (1,604/282,692).

### Tumor analysis.

**Figure 2:**
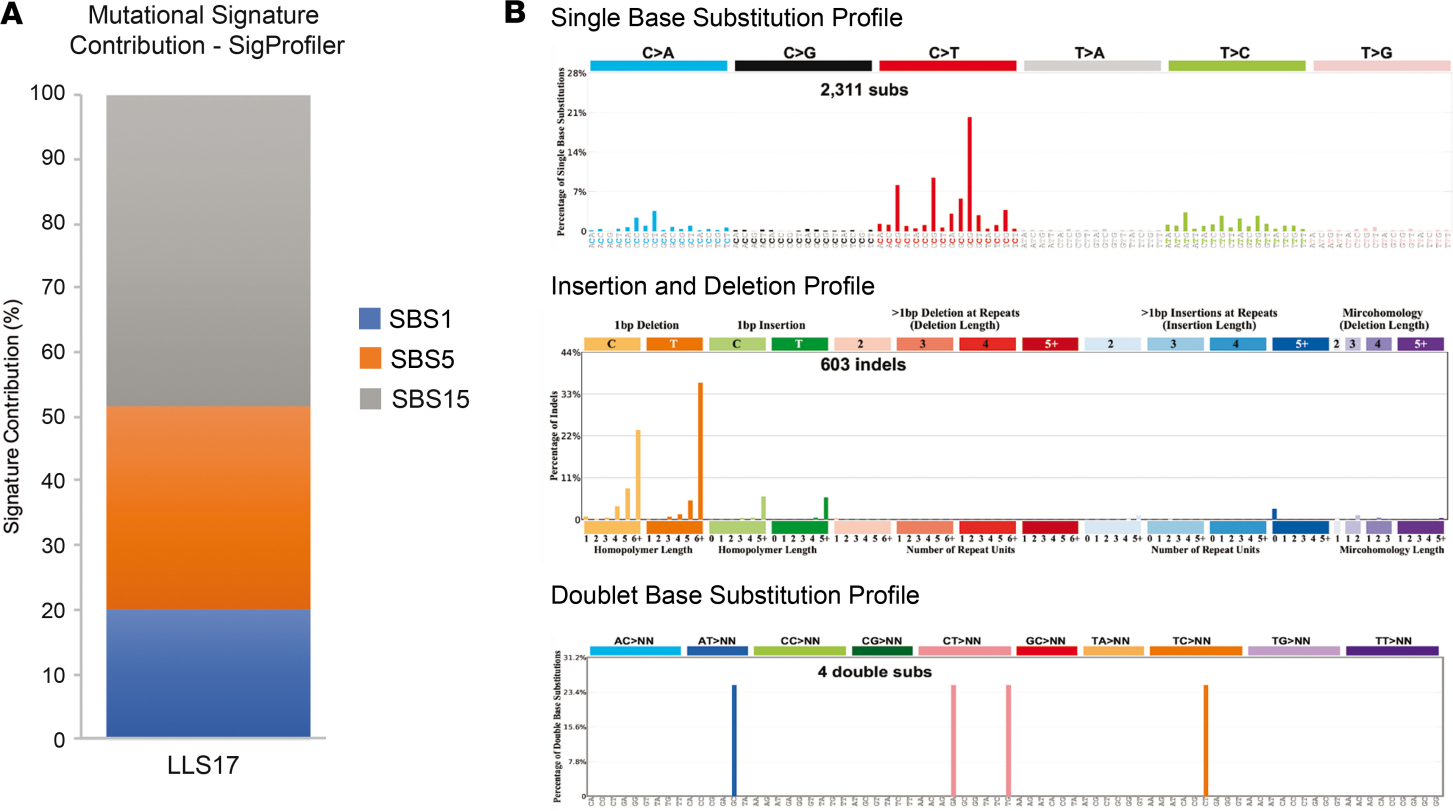
Tumor mutational profiling analysis. (**A**) Mutational signatures contribution extracted with SigProfiler of the LLS17 tumor sample showing a significant contribution of the SBS15 signature, associated with defective MMR system. (**B**) Single base substitution, insertion and deletion, and doublet base substitution profiles. LLS, Lynch-like syndrome; SBS, single-base signature; MMR, mismatch repair.

**Figure 3:**
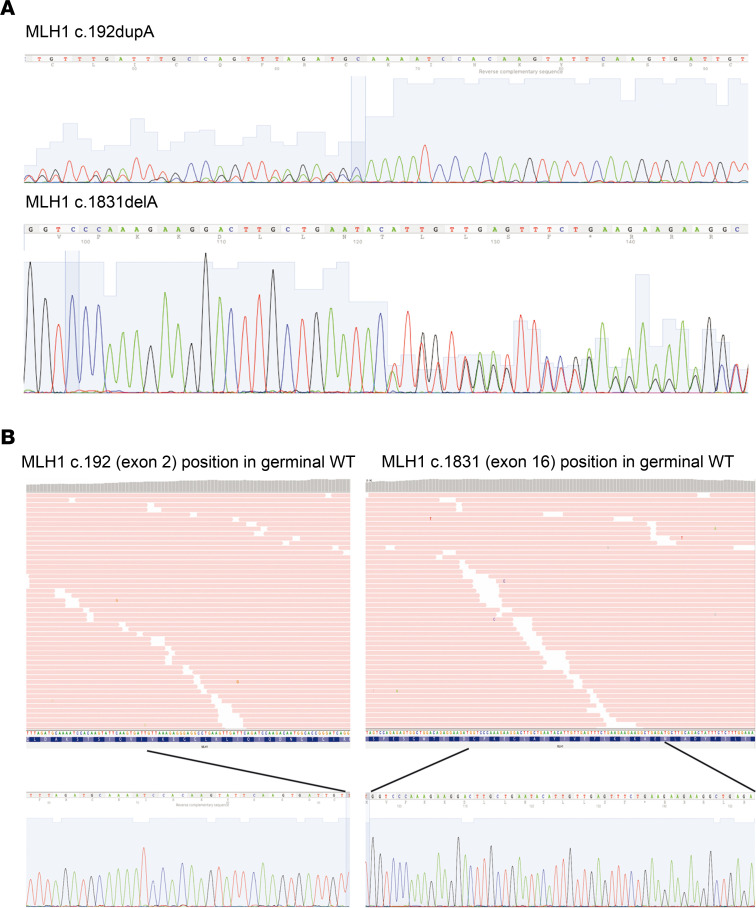
Tumor *MLH1* genetic variants. (**A**) Sanger validation of *MLH1* somatic variants found in LLS17 tumor. For the c.192dupA variant, the reverse complementary sequence is shown. (**B**) Screenshot of IGV manual curation showing germline location of the position of *MLH1* somatic variants, discarding mosaicism. In the lower panel, Sanger chromatogram confirming the absence of both *MLH1* variants in germline DNA. LLS, Lynch-like syndrome; IGV, Integrative Genomics Viewer.

Previous tumor analysis in LLS17 showed an MSI-positive phenotype with loss of MLH1/PMS2 protein expression (Supplemental Figure 1; supplemental material available online with this article; https://doi.org/10.1172/jci.insight.140698DS1), WT *BRAF* V600E, and no *MLH1* somatic hypermethylation. Somatic WES revealed a high tumor mutational burden (77 single nucleotide variants/Mb), an important contribution of the MMR-related mutational signature SBS15 (Figure 2), and provided a set of relevant somatic variants, which are summarized in Supplemental Table 1. Among them, 2 somatic *MLH1* truncating variants (c.129dupA and c.1831delA) were detected and subsequently confirmed by Sanger sequencing (Figure 3A). A putative mosaicism for these variants was disregarded in the patient by manual inspection of the germline WES data at the corresponding genomic locations (Figure 3B).

### CRISPR/Cas9 MCM8^KO^ modeling.

**Figure 4:**
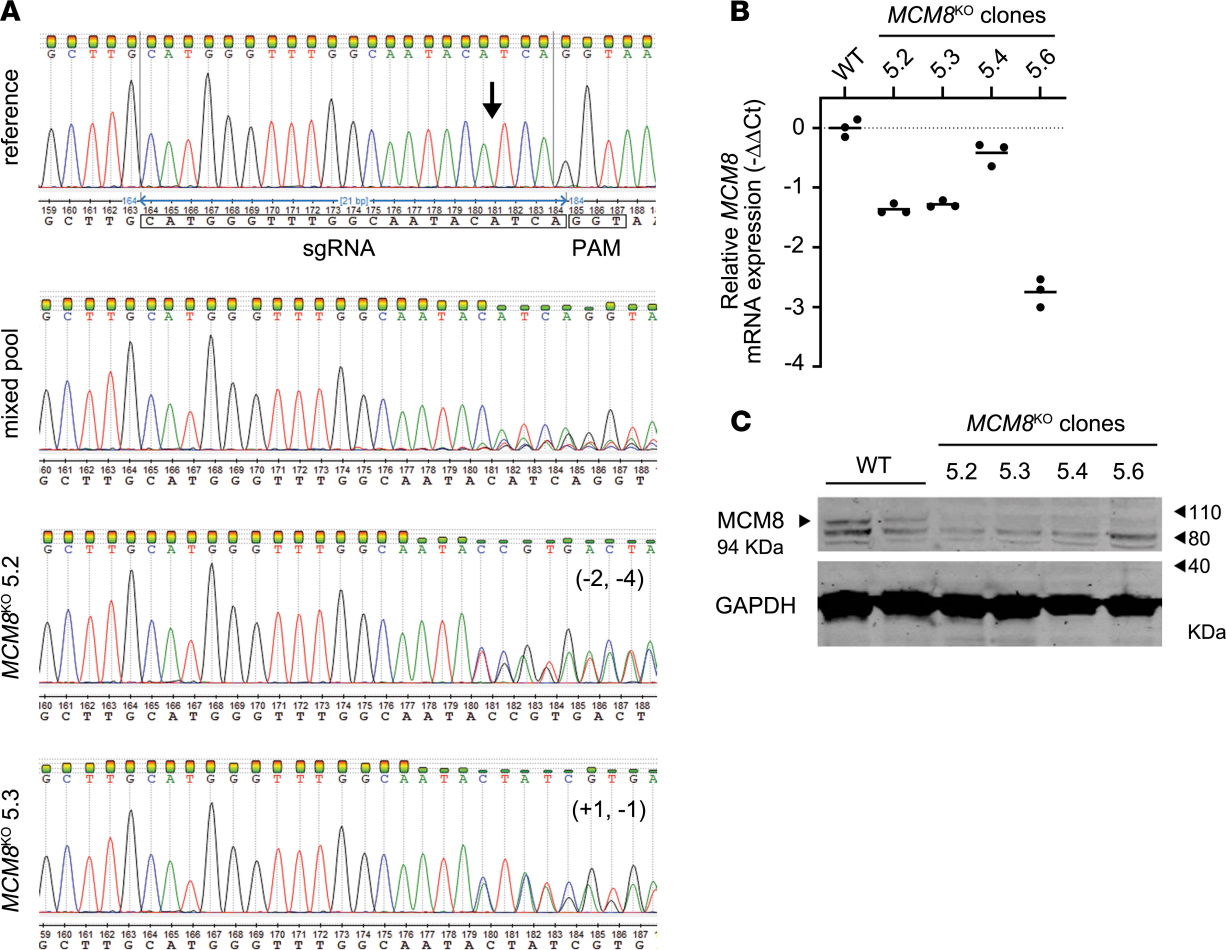
CRISPR/Cas9 *MCM8*^KO^ modeling. (**A**) Sanger sequencing confirmed a disruption in exon 5 of the WT *MCM8* sequence. The black arrow indicates the Cas9 cleavage site, which is located 3 nucleotides upstream the PAM sequence. The Cas9 cleavage efficiency was validated in the mixed pool of cells 2 days after transfection. Gene editing in clones 5.2 and 5.3. was also validated by Sanger sequencing. (**B**) A reduction of *MCM8* RNA expression levels was detected in *MCM8*^KO^ clones when compared *MCM8^WT^* cells (WT), according to real-time PCR results. Samples were assayed in triplicate (*n* = 1). The solid line represents the mean value. (**C**) Immunoblotting analysis of *MCM8*^KO^ protein extracts showed a loss of expression within MCM8 weight range (94kDa). Representative blot of *n* = 2. See complete unedited blots in the supplemental material. For both real-time PCR and Western blot, GAPDH was used as an internal constitutive control. PAM, protospacer adjacent motif.

We established CRISPR/Cas9 *MCM8* knockouts (*MCM8^KO^)* on DLD-1 cells (human CRC cell line) to test the functional impairment of the identified variants and further validate their suspected role in CRC germline predisposition. According to bioinformatic CRISPR prediction tools, a sgRNA targeting the fifth exon was selected. The disruption of *MCM8* and the purity of the clones *were* confirmed by Sanger sequencing. *MCM8* expression depletion was verified at both RNA and protein levels (Figure 4). Two *MCM8*^KO^ clones (5.2 and 5.3) were selected to carry out further expansion and functional characterization.

### Functional characterization of germline variants.

**Figure 5:**
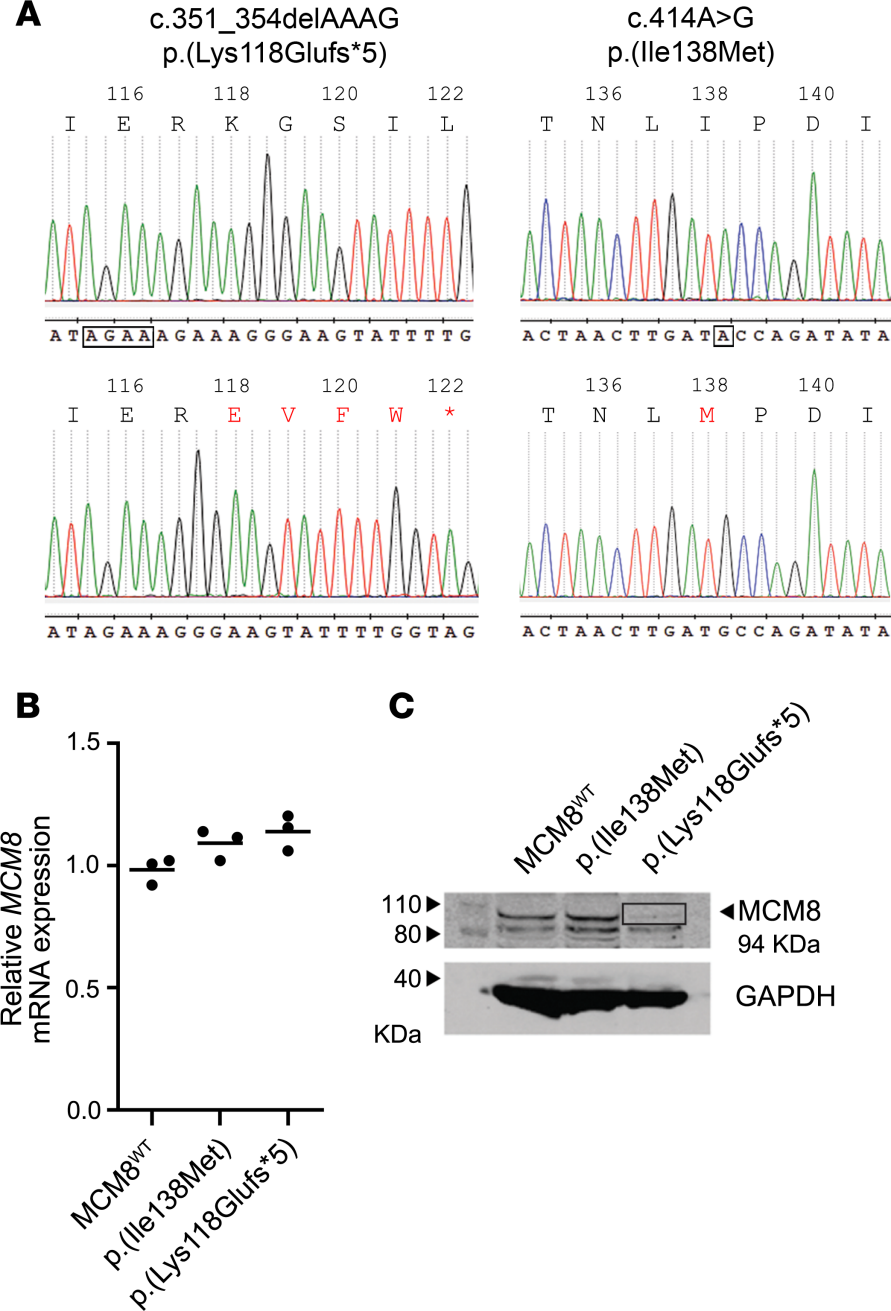
*MCM8* variant generation and expression in *MCM8*^KO^ cells. (**A**) Sanger sequencing of site-directed mutagenesis products confirmed the correct generation of *MCM8* p.(Lys118Glufs*5) (c.345delAGAA) and p.(Ile138Met) (c.414A>G) variants. Changes in the nucleotide sequence are marked, and the altered amino acid sequence is highlighted in bold. (**B**) mRNA expression and (**C**) protein levels of both MCM8 variants and the MCM8^WT^ rescued phenotype in the *MCM8*^KO^ 5.2 clone. In **B**, samples were assayed in triplicate (*n* = 1). The solid line represents the mean value. In **C**, Western blot analysis of MCM8 expression showed no band within the range of MCM8 weight (94 kDa) in the p.(Lys118Glufs*5) *MCM8*^KO^ transfected cells, suggesting a knockout-like pattern. Representative blot of *n* = 3. See complete unedited blots in the supplemental material. GAPDH was used as internal constitutive control.

To evaluate the functional effect of the identified *MCM8* variants, site-directed mutagenesis was performed on a vector carrying the WT ORF of this gene. Both p.(Lys118Glufs*5) and p.(Ile138Met) variants were generated and confirmed by Sanger sequencing (Figure 5A). Both vectors carrying the selected variants were transiently transfected in *MCM8^KO^* cells and their expression was detected at the mRNA level (Figure 5B). However, cells expressing the *MCM8* p.(Lys118Glufs*5) variant showed absence of MCM8 protein expression, suggesting its depletion (Figure 5C).

**Figure 6:**
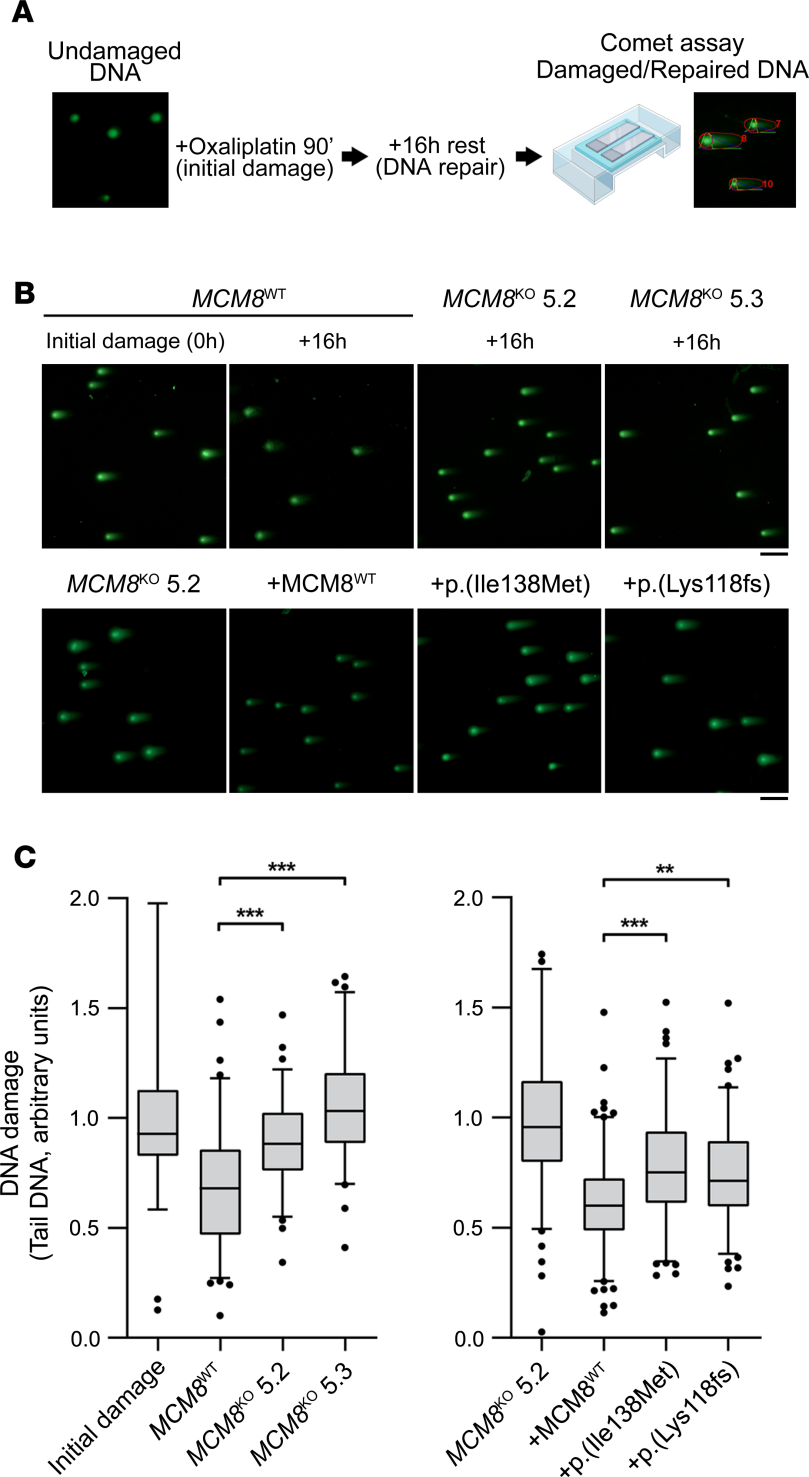
*MCM8* genetic variants displayed less ability to repair damaged DNA. (**A**) Overview of the DNA repair experiment (comet assay). (**B**) Representative images of neutral comet assay (*n* = 3). Upper panel, DNA damage impairment detected in *MCM8*^KO^ cells in comparison with *MCM8*^WT^cells. Lower panel, *MCM8*^KO^ 5.2 expressing p.(Ile138Met) or p.(Lys118Glufs*5) (short format is displayed). MCM8 proteins showed lower DNA repair capacity than the rescued phenotype (MCM8^WT^). Scale bar: 100 μm. (**C**) Quantitative analysis of DNA damage in the 3 independent experiments, measured as the amount of tail DNA. Box-and-whisker plots represent 25th to 75th and 5th to 95th percentiles, respectively. The solid line represents the median value. **P < 0.01, ***P < 0.001, 1-way ANOVA with Tukey’s post hoc test.

Because MCM8 is involved in the repair of DNA double-strand breaks (DSBs), the comet assay was used to monitor DNA damage (Figure 6A). Experimental conditions were set up by a time course of DNA repair kinetics on *MCM8*^WT^ and *MCM8*^KO^ cells. After oxaliplatin treatment, cells were allowed to recover during different time points. DNA repair was already noticeable in *MCM8*^WT^ cells after a 16-hour resting period, whereas at the same time point, *MCM8*^KO^ 5.2 and 5.3 clones still showed sustained DNA damage (Supplemental Figure 2A). This 16-hour resting period was selected to further characterize the repair activity of *MCM8* variants through the comet assay. Results revealed that *MCM8*^WT^ cells almost completely recovered DNA integrity 16 hours after treatment, as they showed less dispersed DNA tail comets. Both *MCM8*^KO^ 5.2 and 5.3 cells showed an increased DNA damage grade after the same resting period, reflecting DNA DSB repair impairment (Figure 6B, upper panel).

Once the implication of *MCM8* on DNA DSB repair was confirmed, we proceeded to test the effect of p.(Ile138Met) and p.(Lys118Glufs*5) variants. To do so, the *MCM8*^KO^ 5.2 clone was transiently transfected with vectors expressing either MCM8^WT^, p.(Ile138Met) or p.(Lys118Glufs*5), exposed to oxaliplatin treatment, and then allowed to recover. We observed that cells expressing both p.(Ile138Met) and p.(Lys118Glufs*5) variants showed larger tails (Figure 6B, lower panel) and therefore a higher DNA damage retention in comparison with the rescued phenotype (MCM8^WT^). The quantitative analysis from the obtained comet images confirmed the impaired DNA repair capacity of MCM8-depleted cells and those expressing both *MCM8* variants (Figure 6C, Supplemental Figure 2B).

### Microsatellite instability and mutational signatures analyses.

**Figure 7:**
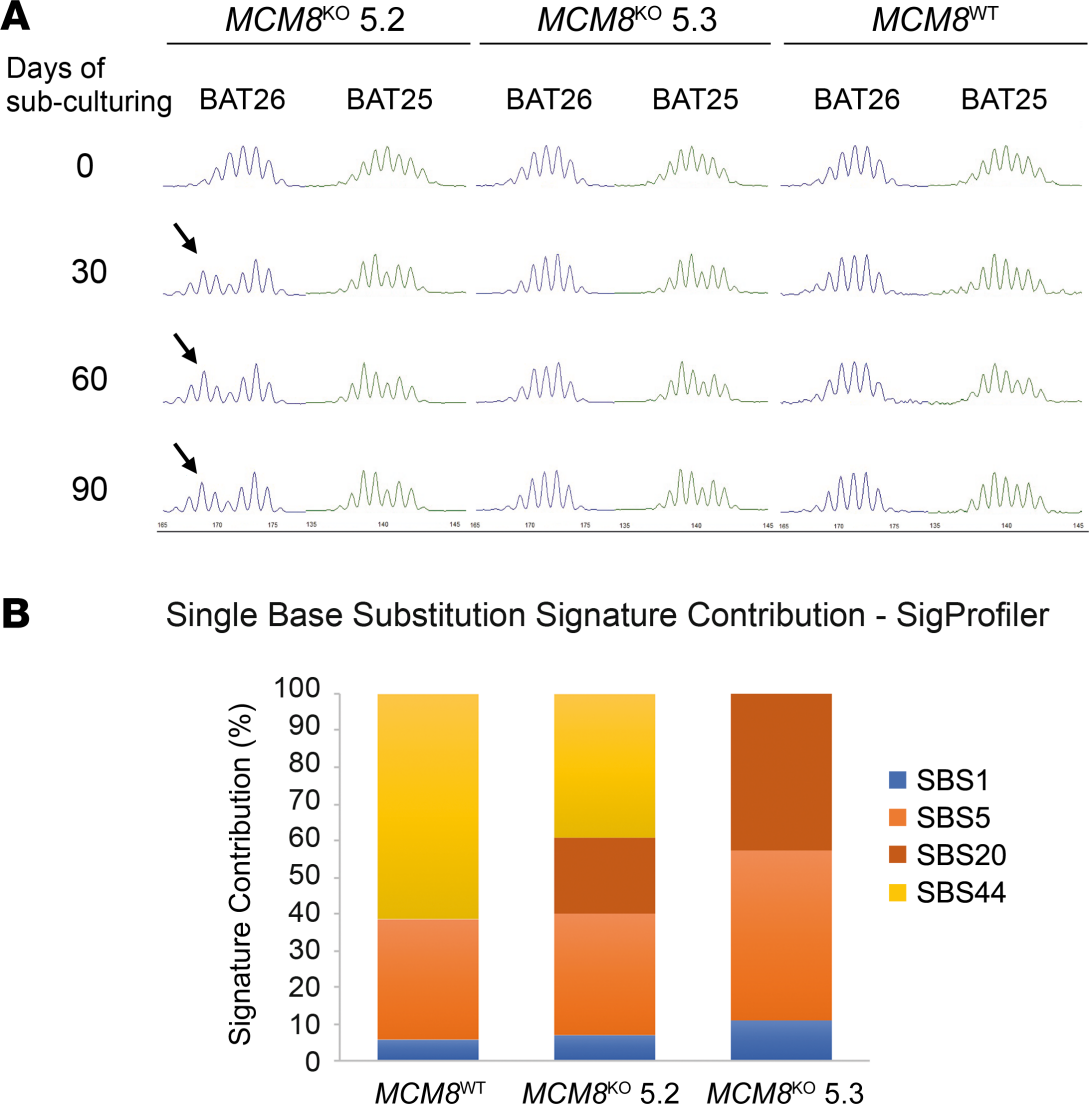
*MCM8* deficiency can initiate MSI and mutational signature related to MSI. (**A**) After 30 days of subculturing, *MCM8^KO^* 5.2 cells already showed a profile shift for BAT25 and BAT26 microsatellite markers when comparing with day 0. This profile was detected throughout the different time points of the culture process. The *MCM8*^KO^ 5.3 clone exhibited milder MSI alterations only apparent for BAT25. Profile shifts are indicated with an arrow. (**B**) *MCM8*^KO^ 5.2 and 5.3 displayed a significant contribution of SBS20 mutational signature (associated with concomitant *POLD1* mutations and defective DNA mismatch repair), whereas it was not detected on *MCM8*^WT^ cells cultured over the same period. MSI, microsatellite instability.

To determine if depletion of *MCM8* was involved in alteration of the MMR system in our model, MSI was analyzed in *MCM8*^WT^, *MCM8*^KO^ 5.2, and *MCM8*^KO^ 5.3 cells after 30, 60, and 90 days of subculturing. When comparing with *MCM8*^WT^, a MSI-like profile was especially evident in the *MCM8*^KO^ *5.2* clone, already present on day 30 of subculturing, whether it was more subtle for the *MCM8*^KO^ 5.3 clone (Figure 7A).

To gain more insight about the relationship between *MCM8* deficiency and the MSI phenotype, we performed WES and mutational signature analysis in *MCM8^WT^*, *MCM8^KO^* 5.2, and *MCM8^KO^* 5.3 cells after 120 days of culturing. We were able to determine which genetic variants appeared during culturing and focus our signature analysis on them. Single-base signatures (SBSs) SBS1, SBS5, SBS20, and SBS44 were detected in the analyzed samples (Figure 7B). SBS1, SBS5, and SBS44 were present in all samples. SBS44 is associated with a defective DNA MMR and was also present in *MCM8^WT^* cells, most likely due to the fact that cells used as cellular model (DLD-1) are MSH6-defective. Interestingly, both *MCM8*^KO^ clones acquired a distinctive, marked contribution of the SBS20 not present in *MCM8^WT^*. This signature also represents one of the 7 signatures associated with a MMR system impairment and it is currently associated at the moment to concurrent *POLD1* pathogenic variants and defective DNA MMR (11). Regarding mutational burden, *MCM8*^KO^ 5.2 cells accumulated more variants than *MCM8*^WT^ or *MCM8*^KO^ 5.3 cells (Supplemental Figure 3A). At the point variant level, no relevant putative somatic mutations were detected by WES in *MCM8^WT^*, *MCM8^KO^* 5.2, and *MCM8^KO^* 5.3 cells after culturing. However, considering the length of indel substitutions, a higher frequency of indels greater than 5 base pairs was found in the *MCM8*^KO^ 5.3 clone, suggesting that other alterations in DNA repair mechanisms, such as homologous recombination (HR) (12), could be a concomitant defect associated to *MCM8* depletion (Supplemental Figure 3B).

### Screening of the candidate gene variants in an independent cohort.

An independent cohort of 131 Dutch unaffiliated familial cancer cases (mainly CRC) with available WES data was accessible. Both *MCM8* and *MCM9* were screened in this cohort because a link between *MCM9* and inherited predisposition to mixed polyposis and early-onset CRC has also been previously suggested and both proteins cooperate to perform their function (13). Results are summarized in Table 1. A patient with breast cancer carried biallelic *MCM8* genetic variants in the context of a breast cancer family, and 5 additional heterozygote carriers were detected. Regarding *MCM9*, 2 families carried biallelic genetic variants, including a LLS patient with premature ovarian failure (POF) and a familial MMR-proficient CRC patient, and heterozygote variants were found in 12 patients. Family trees for biallelic carriers are available in Supplemental Figure 4. Mostly, additional segregation for the detected genetic variants was not possible in these families, with the exception of one of the families that carried biallelic *MCM9* variants, where an affected brother of the proband also carried both variants. However, according to an in silico prediction (CADD >15), most of them could correspond to potentially pathogenic genetic variants.

## Discussion

We analyzed 16 patients with early-onset nonfamilial, nonpolyposis CRC and LLS to identify germline candidate genes in this scenario. Although biallelic somatic pathogenic variants in MMR genes may account for over half of CRC labeled as LLS (10), some patients with LLS may have a hereditary origin, especially in the early-onset setting, such as biallelic *MUTYH* or monoallelic *POLE*/*POLD1* carriers (14). We identified 2 potentially pathogenic germline biallelic *MCM8* variants in one of these patients, who also presented somatic biallelic *MLH1* mutations. Therefore, our main hypothesis was that germline alterations in *MCM8* following a recessive pattern of inheritance could be involved in predisposing to CRC with a somatic MMRd phenotype.

The minichromosome maintenance 8 homologous recombination repair factor (*MCM8*) gene raised interest as a candidate due to its previous association with ovarian/gonadal failure and chromosomal instability (15). Also, it forms a complex with *MCM9*, a gene required in the MMR system (16) and recently associated with a germline predisposition to CRC as well as with recessive pattern of inheritance (13). MCM8 works together with MCM9 in a helicase hexameric complex involved in several functions such as genome maintenance, meiotic recombination, and DSB repair via HR (17). Although germline variants in several DNA helicases are associated with cancer (18) and recessive *MCM9* variants are predisposed to CRC and POF in a recessive manner (13), there is currently no evidence of an association between *MCM8* and germline CRC predisposition. Remarkably, germline alterations in this gene have been related to POF and chromosomal instability (19, 20). Very recently, the same *MCM8* truncation variant found in LLS17 in this study p.(Lys118Glufs*5) is found in homozygosis in 2 sisters affected with POF (21).

MCM8 participates in DSB repair by HR interacting with MRN complex (MRE11-RAD50-NSB1) and is required for nuclease activity and stable association with DSBs (22). Cells lacking MCM8 or MCM9 are viable but highly sensitive to interstrand cross-linking–inducing (ICL-inducing) agents and exhibit more chromosome aberrations in the presence of mitomycin C compared with WT cells (15). During ICL repair, MCM8 and MCM9 form nuclear foci that partly colocalize with Rad51. MCM8-9 dimer also works downstream of the Fanconi anemia and the BRCA2/Rad51 pathways and is required for HR that promotes sister chromatid exchanges, as a hexameric ATPase/helicase (23). Although only *MCM8* variants located in the helicase domain are tested to explore HR deficiency (23), we showed to some extent that the *MCM8* p.(Ile138Met) variant, located in the DNA binding domain, can impair DSB repair capacity.

By performing *MCM8* gene editing in a cellular model and ectopic reintroduction of the identified genetic variants, we were able to demonstrate its plausible effect on DNA repair efficiency. Besides, although several genes implicated in DNA repair are spotlighted as probable causes of the underlying mechanisms of LLS CRC (24), this study differs from previous reports in the fact of performing a thorough functional characterization of the proposed candidate gene. Additionally, although HR and MMR systems are inextricably linked (25), we were capable of suggesting that both systems could be impaired by biallelic pathogenic variants in *MCM8*.

**Figure 8:**
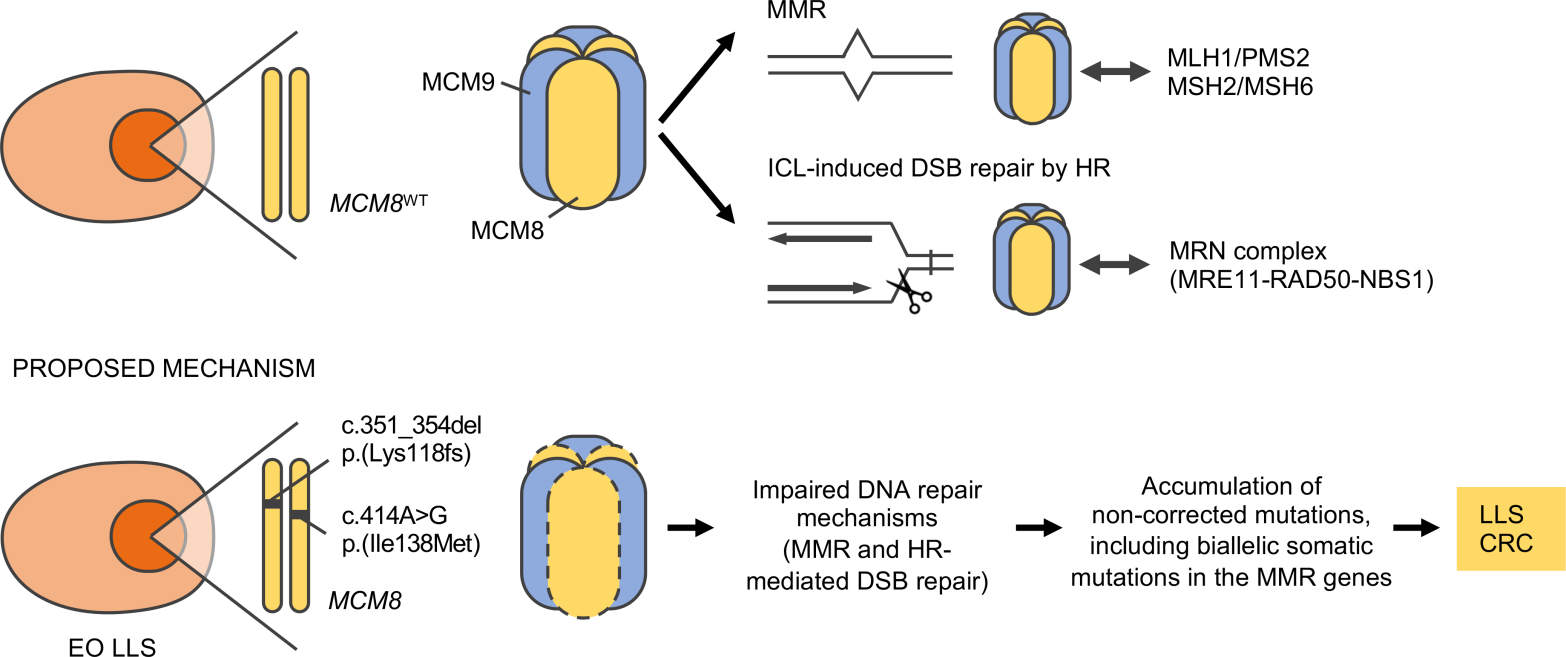
Proposed molecular mechanism of pathogenicity for *MCM8* variants. The MCM8/MCM9 hexameric complex is required for the MMR system and the HR-mediated DNA repair caused by ICL agents. Previous work has demonstrated that MCM9 interacts with the MMR proteins and is recruited to the mismatch lesion (16). Also, the MCM8/MCM9 complex is required for the MRN protein complex at DNA damage foci to facilitate DNA resection, a key step on DNA DSB repair (22). We propose that biallelic germline mutations in *MCM8* affect the MCM8/MCM9 protein complex, impairing both DNA repair pathways and leading to the accumulation of noncorrected mutations. These alterations, in some cases, can also promote somatic biallelic MMR inactivation and LLS. CRC, colorectal cancer; DSBs, double-strand breaks; EO LLS, early-onset Lynch-like syndrome; HR, homologous recombination; ICL, interstrand cross-linking; MMR, mismatch repair.

However, the relationship between MCM8 and the MMR system still remains unclear. MCM9 is required for mammalian MMR (16), as it coimmunoprecipitates with MCM8, MSH2 and MLH1 (both key MMR system proteins), and its deficiency is linked to MSI and a MMR alteration. MCM8 and MCM9 arose early during eukaryotic evolution and are conserved among many eukaryotic organisms (not in yeast or *C*. *elegans*). *D*. *melanogaster* and related species possess only the MCM8 protein (i.e., REC) and MCM9 is absent (26). Additionally, MCM8 protein expression is lost in *MCM9* knockout cells, suggesting that both proteins are needed for the formation of an hexameric complex and its stabilization (16, 23). Thus, this fact agrees with variants in *MCM8* and *MCM9* being responsible for similar phenotypes as those previously reported (13). However, MCM8 or MCM9 defects may not always be linked with a MMR-deficient or MSI-positive tumor. In fact, the MMR system was preserved and MSI was negative in the patient with biallelic *MCM9* CRC previously reported (Y. Goldberg and E. Pikarsky, unpublished data). On the other hand, the developed *MCM8*^KO^ model showed impairment of the MMR system. Additionally, *MCM8*^KO^ cells developed the SBS20 mutational signature over time. This signature is associated with MSI CRC and concomitant *POLD1* somatic mutations (11, 27). It represents a mutational profile that reflects the biological interaction between *POLD1* and the MMR system rather than the sum of both processes. It remains to be elucidated whether the interaction between MCM8 and the MMR system represents a similar profile. Based on these results, we hypothesize that *MCM8* pathogenic variants may perturbate the function of the MCM8/MCM9 hexameric complex, impairing both the MMR and the HR-mediated DNA repair pathways (Figure 8).

Finally, the presence of *MCM8*/*MCM9* genetic variants in the independent analyzed cohort with a putative involvement in familial cancer/CRC predisposition supports, to some extent, our conclusions. Besides, as highlighted by the biallelic *MCM8* carrier with breast cancer, a potential pleiotropy effect for this gene could be hypothesized to include germline predisposition to breast cancer besides CRC. Further family segregation and functional characterization of these variants are warranted, as well as replication in additional familial CRC cases to confirm the implication of these genes in hereditary CRC and other neoplasms.

To conclude, with our study we provide evidence that some LLS CRC cases with a defective MMR system, showing somatic biallelic MMR inactivation, may be caused by underlying germline pathogenic variants in CRC predisposing genes, especially in patients with an early onset. We suggest *MCM8* as a potential CRC predisposing gene following a recessive inheritance pattern, and recommend this gene and *MCM9* to be included in future screening of unaffiliated familial CRC cohorts to gain additional knowledge of its involvement in germline CRC predisposition.

## Methods

### DNA extraction.

Germline, tumor, and cultured cells’ DNA were extracted with the QIAamp DNA Blood, QIAamp DNA FFPE, and QIAamp DNA Mini Kits, respectively (QIAGEN) following the manufacturer’s instructions.

### Exome sequencing.

Germline and tumor WES were performed in DNA samples using the HiSeq2000 platform (Illumina) and SureSelectXT Human All Exon v5 kit (Agilent) for exon enrichment at Centre Nacional d’Anàlisi Genòmica (https://cnag.crg.eu). Indexed libraries were massively parallel sequenced using a paired-end 2 × 75 bp read length protocol. Sequencing data quality control previous to its analysis was performed in all samples using the Real-Time Analysis software sequence pipeline (Illumina). The Burrows–Wheeler Aligner (BWA-MEM algorithm) was used for the human reference genome read mapping (build hs37d5, based on NCBI GRCh37) (28).

### Sequencing data analysis.

The GATK HaplotypeCaller tool was used for single nucleotide variants (SNVs) and short indels calling in germline samples, and GATK MuTect2 and Strelka2 were applied in tumor samples using previously developed R language in-house pipelines (29–32). Several databases were evaluated for variant annotation, including SnpEff and dbNSFP. PhyloP, SIFT, PolyPhen2, MutationTaster, LRT, and CADD were used for missense variants pathogenicity prediction as previously described (30, 33). For germline DNA data, we selected those genes following a recessive pattern of inheritance with 2 potentially pathogenic variants per individual, both presenting an allele frequency in the Genome Aggregation Database (gnomAD, https://gnomad.broadinstitute.org/) lower than 1%. Guidelines for the interpretation of sequence variants from the American College of Medical Genetics and Genomics and the Association for Molecular Pathology were also used (34). STRUM was used for predicting the effect of genetic variants regarding fold stability change of protein molecules (35). Tumor variants were restrained to those having a coverage of greater than or equal to 10X in both germline and somatic samples, having an alternative allelic frequency in the tumor of greater than or equal to 20%, and being truncating or missense variants fulfilling at least 3 of the missense pathogenicity tools criteria. SigProfiler (11, 36) was used to perform SNV mutational signature refitting analysis according to COSMIC reference signatures (37). Tumor mutational burden was assessed exploring somatic SNVs and indels.

Additional germline variant prioritization was carried out to select those actually relevant for the CRC phenotype and then were manually curated using the Integrative Genomics Viewer (38). Germline and tumor candidate variants were subsequently validated by Sanger sequencing. Primer details are available in Supplemental Table 2.

### Human CRC cell line.

The DLD-1 human CRC cell line was purchased from the American Type Culture Collection and cultured in RPMI-1640 medium and supplemented with 10% FBS (Gibco, Thermo Fisher Scientific) at 37°C in 5% CO_2_.

### Plasmids.

LentiCRISPRv2-Puro (98290, Addgene) expression vector was available. *MCM8* ORF (NM_032485.5) cloned into the pcDNA3.1 expression vector (OHu10568D) was purchased from GenScript.

### CRISPR/Cas9-mediated MCM8 knockout generation.

The Benchling (http://benchling.com) CRISPR tool was used to design the single guide RNA (sgRNA) against the coding region of the *MCM8 gene*. The sgRNA was cloned into the LentiCRISPRv2-Puro vector and transiently transfected into the DLD-1 CRC cell line. Two days later, transfected cells were puromycin-selected (4 μg/mL) and seeded into a 96-well plate at a density of 1 cell/well. Several clones were characterized and selected for further analysis. *MCM8* gene editing was validated by Sanger sequencing, and gene downregulation and depletion were checked by quantitative real-time PCR and Western Blot, respectively.

### Antibodies.

Polyclonal antibody against MCM8 (ab183045) was from GeneTex. Anti-GAPDH (14C10) was purchased from Cell Signaling. Goat anti-rabbit (SA5-10036) DyLight 800 secondary antibody was acquired from Thermo Fisher Scientific.

### RNA extraction and quantitative real-time PCR.

Total RNA extraction was performed with the RNeasy Mini Kit according to manufacturer’s instructions (QIAGEN). RNA was retrotranscribed using the Applied Biosystems High-Capacity cDNA Reverse Transcription Kit (Thermo Fisher Scientific). Multiplex quantitative PCR was performed with the Applied Biosystems 7300 PCR System by using a specific TaqMan assay for *MCM8*-FAM (hs01067422_m1). The endogenous control gene was *GAPDH*-VIC (4326317E). Relative expression levels of each target gene were calculated for each sample as –ΔCt values (–ΔCt= – [Ct target gene – Ct endogenous control]).

### Protein extraction and Western blot.

To obtain whole-cell protein extracts, cells were detached from cell culture plates with Accutase (MilliporeSigma) and lysed with RIPA buffer and supplemented with cOmplete Protease Inhibitor Cocktail and PhosSTOP (Roche). Sample protein concentrations were determined by using the Pierce BCA Protein Assay Kit (Thermo Fisher Scientific). Equal amounts of protein lysates were resolved in NuPAGE Bis-Tris protein gel electrophoresis followed by the transfer of protein onto Immobilion PVDF membranes (Millipore) according to manufacturer’s protocols (Thermo Fisher Scientific). Proteins were blotted with the indicated primary and secondary Dylight antibodies and detected by using the Odyssey Imaging System (LI-COR). See complete unedited blots in the supplemental material.

### Site-directed mutagenesis.

The Q5 Site-Directed Mutagenesis Kit (NEB) was used to introduce the variants of interest in the WT pcDNA3.1-*MCM8* expression vector. Mutagenic primers were designed using the NEBaseChanger tool and obtained from IDT (Coralville) (Supplemental Table 2). Mutagenesis products were verified by Sanger sequencing.

### Microsatellite instability assay by multiplex PCR.

*MCM8*^WT^ and *MCM8*^KO^ cells were maintained during 90 days. DNA from cells was extracted every 30 days to test MSI profiles in 2 different microsatellite markers (BAT25, BAT26) (39) by using a capillary electrophoresis genetic fragment analyzer (Applied Biosystems). Primer sequences are available in Supplemental Table 2.

### Neutral single-cell gel electrophoresis (Comet assay).

*MCM8*^KO^ clones were transiently transfected with WT or mutated p.(Lys118Glufs*5) or p.(Ile138Met) plasmids using X-tremeGENE HP DNA transfection reagent. Two days later, cells were subjected to selection with 1 mg/mL of G418 (InvivoGen) for 72 hours, as the *MCM8* expression pcDNA3.1 vector carries a neomycin resistance cassette.

To induce DNA damage, cells were incubated in media containing 100 μM oxaliplatin for 90 minutes. After a resting period of 16 hours, cells were collected to evaluate their DNA repair capacity by a neutral comet assay (Trevigen). As a control, oxaliplatin-treated cells with no resting period were also collected.

### Exome sequencing and mutational signatures in MCM8^KO^ cell lines.

Three cell lines (*MCM8^WT^* cells and 2 different *MCM8^ko^* clones) were cultured during 120 days to allow mutation accumulation. Then DNA samples were obtained and evaluated by WES together with a DNA sample from the original *MCM8^WT^* pool (day 0) to assess the initial mutational background. Data were processed as previously described (see above). Mutect2, VarScan, and Strelka2 were used for variant calling (29, 31, 40). Only the variants that were called by at least 2 of these 3 callers were chosen for further analysis. Results from *MCM8^WT^* cells at the initial time point (0 days) were used as parental normal control against the 3 cells lines cultured for 120 days to exclude the initial mutational background and only consider genetic variants that appeared during culturing. SigProfiler was used again as previously described (11, 36).

### Screening of the candidate gene variants in an independent cohort.

To seek additional patients with potentially pathogenic germline variants in our candidate gene, access to WES data from an independent cohort of 131 Dutch unaffiliated familial cases (mainly CRC) was granted for replication purposes. We selected coding potentially pathogenic variants (missense, frameshift, truncating, splicing altering, CADD > 15), presenting an allele frequency in gnomAD lower than 1%.

### Statistics.

For multiple comparisons, 1-way ANOVA was followed by Tukey’s multiple comparisons test. A *P* value less than 0.05 was considered significant.

### Study approval.

Sixteen patients with nonpolyposis LLS CRC diagnosed before the age of 40 were selected from a cohort previously described by Antelo et al. (6) and from the high-risk clinic for gastrointestinal cancer at Hospital Clínic in Barcelona. These patients presented tumors with MSI and/or IHC loss of MLH1, MSH2, MSH6 or PMS2, WT *BRAF* V600E and/or negative *MLH1* methylation, and with no germline pathogenic variants in the MMR or *EPCAM* genes. This study was approved by the research ethics committee of Hospital Clínic in Barcelona (2011/6440), and written informed consent was obtained in all cases.

## Author contributions

MG, LB, MA, and SCB conceived the study. MA, SCB, AC, and FB acquired funding. MG, LB, JGAO, MDG, JM, MC, TO, SI, GM, DC, SAS, MN, TVW, YG, EP, JR, ER, MA, and SCB provided investigation. MG, LB, JGAO, MDG, JM, MC, TO, SI, GM, DC, SAS, MN, TVW, YG, EP, JR, ER, AC, FB, MA, and SCB obtained resources. LB, MA, and SCB supervised this study. MG, LB, JGAO, MDG, JM, MC, TO, SAS, MN, TVW, YG, EP, MA, and SCB visualized data. MG, LB, MA, and SCB wrote the original draft. All authors reviewed and edited the manuscript.

## Supplementary Material

Supplemental data

## Figures and Tables

**Table 1:**
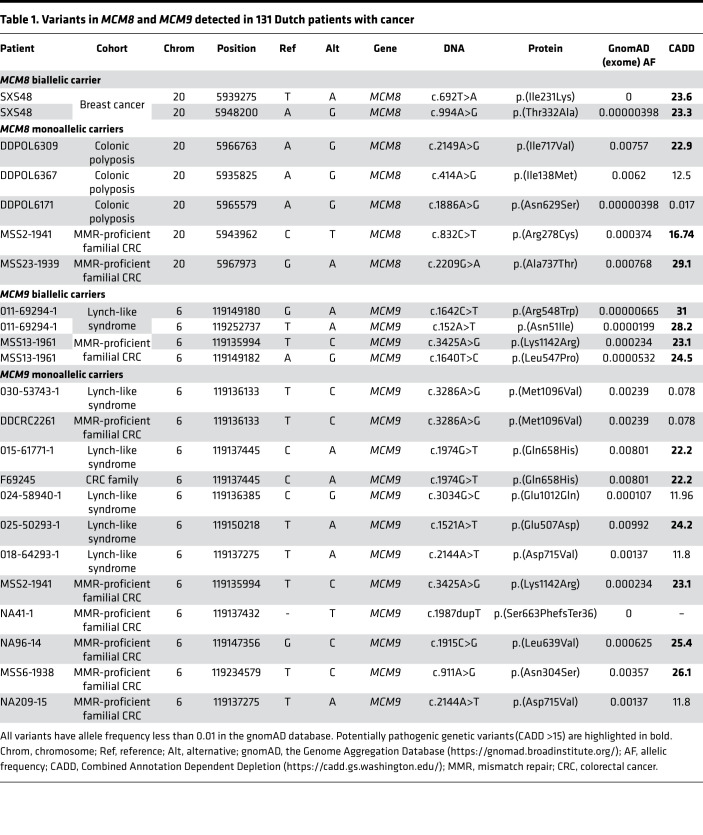
Variants in *MCM8* and *MCM9* detected in 131 Dutch patients with cancer

## Version 1 08/25/2020

In-Press Preview

## Version 2 09/17/2020

Electronic publication
