# Supplementary material for: Germline biallelic Mcm8 variants are associated with early-onset Lynch-like syndrome
Source: JCI Insight. 2020 Sep 17;5(18):e140698. doi: 10.1172/jci.insight.140698 (PMC7526538; doi:10.1172/jci.insight.140698)
Supplement: Supplemental data [file jciinsight-5-140698-s239.pdf]

SUPPLEMENTAL PDF

Supplementary Figure S1. (**A**) Microsatellite Instability of the LLS17 with BAT26, BAT25, NR21, NR24 and MONO27 markers showing a MSI-H phenotype. (**B**) Immunohistochemical testing for MMR proteins demonstrating MLH1/PMS2 loss.

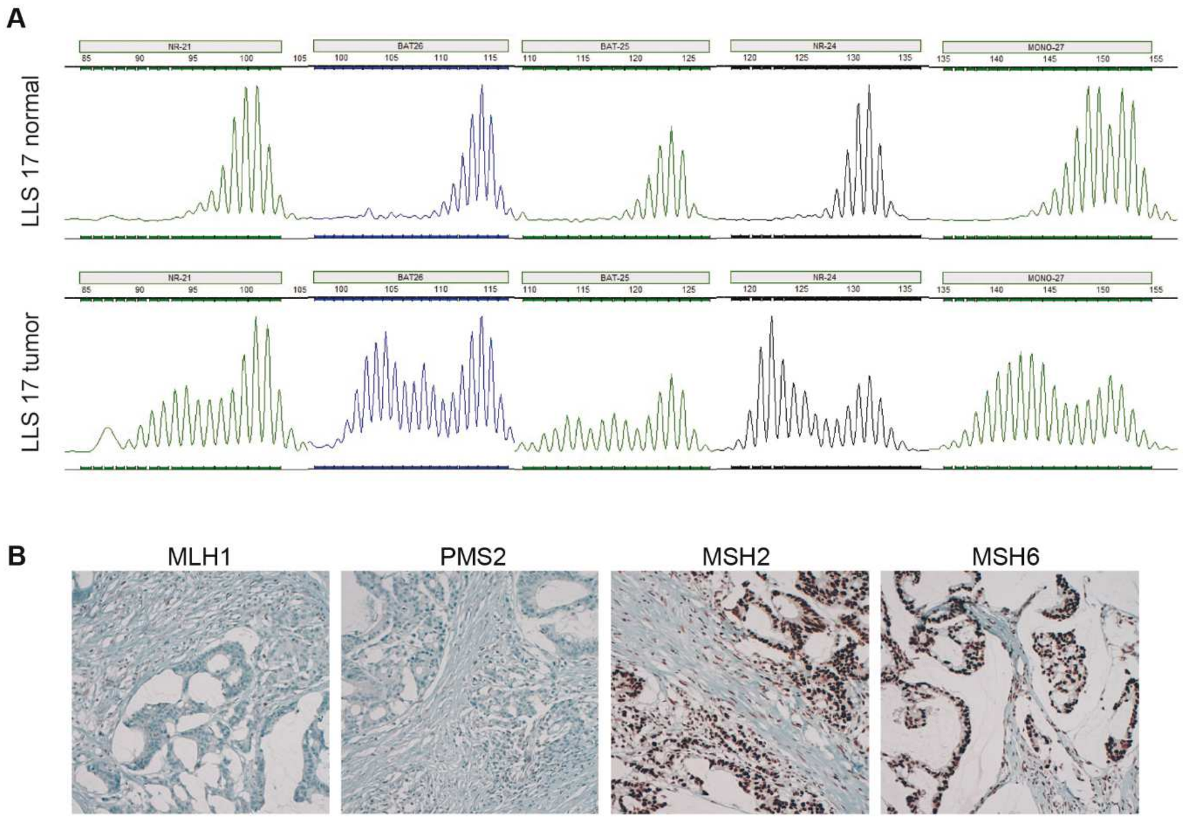

Supplementary Figure S2. (**A**) DNA electrophoresis of a time-course kinetics on cells after 100 µM of oxaliplatin treatment (*n* = 1). *n.t.,* no treatment. (**B**) Tail Area and Tail Intensity parameters measured in the three independent experiments of the comet assay. The effect of MCM8 depletion is displayed in both *MCM8*^{KO} 5.2 and 5.3, as well as the effect of both p.(Lys118Glufs*5) and p.(Ile138Met) variants in the *MCM8*^{KO} 5.2 clone. Box and whiskers represent 25–75 and 5–95 percentiles, respectively. The solid line represents the median value. *P<0.05, ***P<0.001, 1-way ANOVA with Tukey post hoc test.

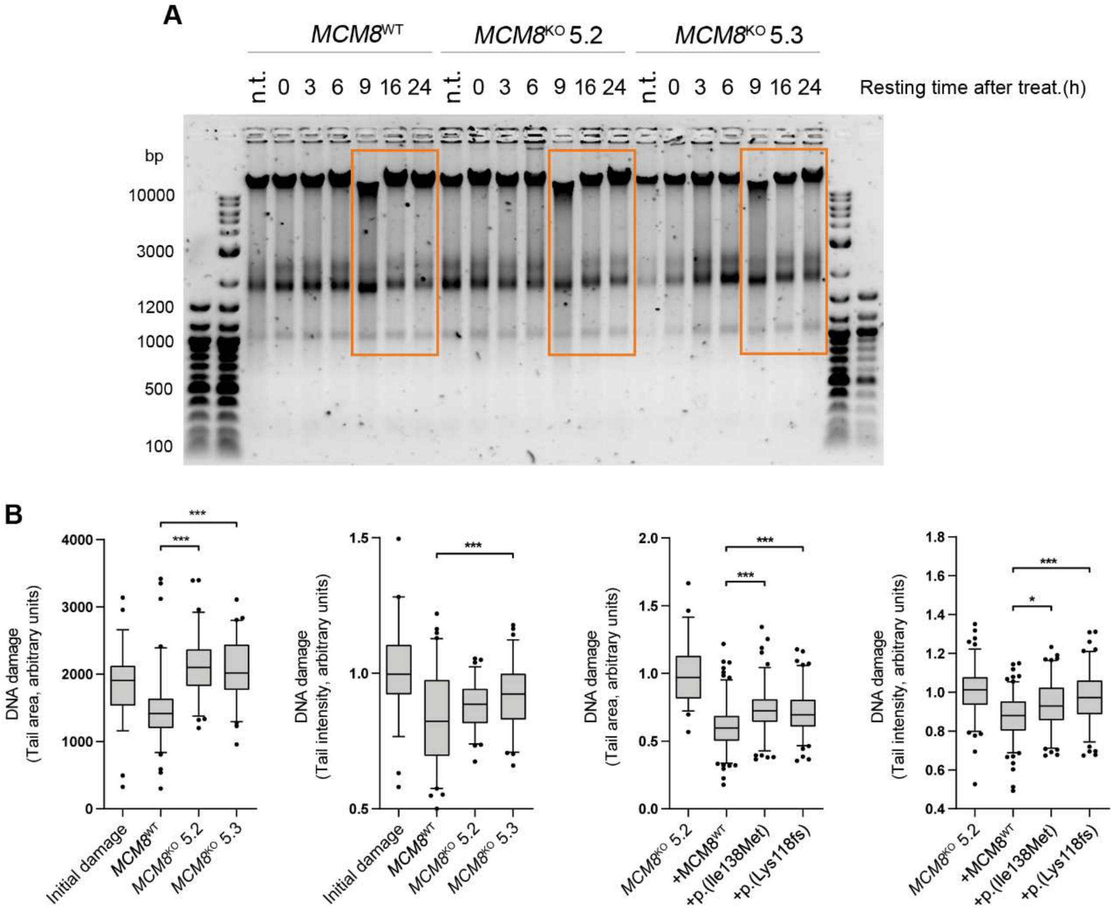

Supplementary Figure S3. (**A**) Exome variant count showed that *MCM8*^{KO} 5.2 accumulated more total variants in 120 days of sub-culturing than *MCM8*^{KO} 5.3 and *MCM8*^{WT}. (**B**) *MCM8*^{KO} 5.3 showed more indels variants longer than 5 base pairs in comparison to *MCM8*^{KO} 5.2 or *MCM8*^{WT}.

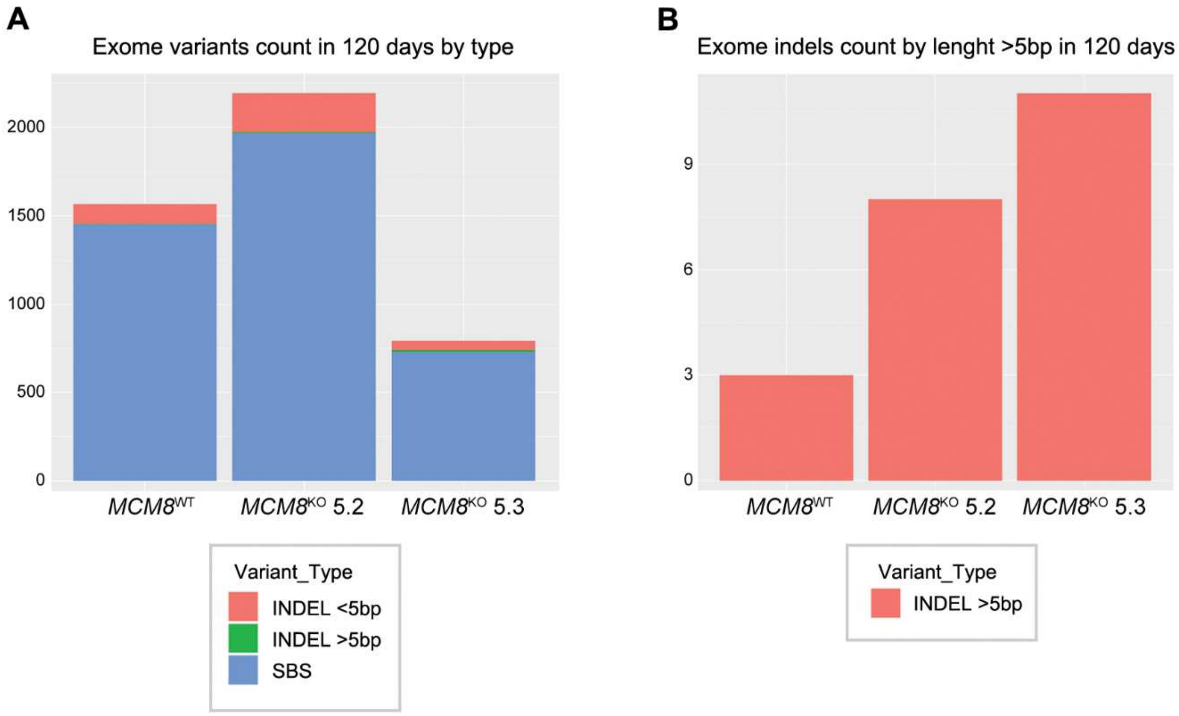

Supplementary Figure S4. Family trees of the Dutch cohort with *MCM8* or *MCM9* biallelic genetic variants. Probands are indicated by an arrow. Filled symbols represent cancer patients. A slash through the symbol depicts an individual who is deceased. The abbreviations under each symbol indicate the age at death (d.) and/or the diagnosis of a malignancy followed by the age at diagnosis. The numbers in brackets represent relatives merged in the pedigree for clarity. Asterisks show carriers of the biallelic variants. If available, mismatch repair status is indicated. MSS, microsatellite stable; MSI-H, microsatellite instability high.

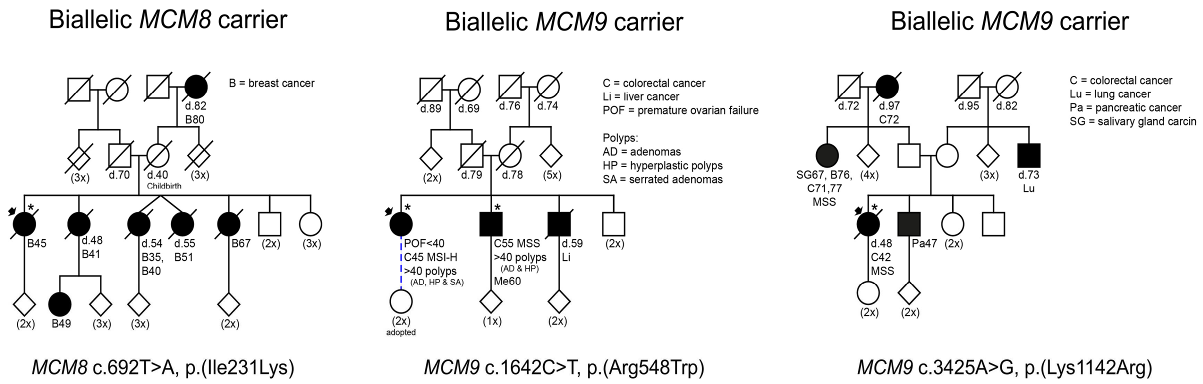

Supplementary Table S1. Relevant tumor variants in the LLS17 patient obtained by ES analysis. Somatic *MLH1* variants are highlighted in bold.

Chrom	Position	Ref	Alt	Gene	DNA	Protein	CADD	Tumor AF	Tumor DP
5	112173847	G	GGA	*APC*	c.2563_2564dupGA	p.(Arg856fs)	--	0,2128	47
17	7578554	A	G	*TP53*	c.376T>C	p.(Tyr126His)	29,3	0,0652	138
11	108124740	C	T	*ATM*	c.2098C>T	p.(Gln700*)	36	0,2407	54
10	89693002	C	CA	*PTEN*	c.491dupA	p.(Val166fs)	--	0,1778	45
10	89717769	TA	T	*PTEN*	c.800delA	p.(Lys267fs)	--	0,2381	21
**3**	**37038118**	**C**	**CA**	***MLH1***	**c.129dupA**	**p.(Ser44fs)**	**--**	**0,2188**	**32**
**3**	**37038118**	**CA**	**C**	***MLH1***	**c.1831delA**	**p.(Ile611fs)**	**--**	**0,3333**	**78**
16	14029458	C	T	*ERCC4*	c.1669C>T	p.(Leu557Phe)	24,8	0,32	50

Chrom, chromosome. Ref, reference. Alt, alternative. CADD, Combined Annotation Dependent Depletion, https://cadd.gs.washington.edu/. DP, coverage depth. AF, alternative allele frequency.

Supplementary Table S2. Primers used in the study.

**Description**	**Forward**	**Reverse**
MCM8 variant validation	GCTCAGTTAATGGTAATTGACTACA	CCTCTCAGTTCTAGCCAACATC
MCM8 sgRNA	CACCGCATGGGTTTGGCAATACATC	aaacGATGTATTGCCAAACCCATGC
lentiCRISPR sgRNA cloning verification	GAGGGCCTATTTCCCATGATT	CCACTCCTTTCAAGACCTAGC
MCM8 c.351_354delAAAG mutagenesis	AGAAAGGGAAGTATTTTGG	ATTTCATCCTTGTCATACAAATC
MCM8 c.414A>G mutagenesis	CTAACTTGATgCCAGATATAGC	TTACTTCACCACCTTCTG
MCM8 ORF sequencing	CAACAAAGACCCCACAGTCA	CCTTGCATGAATATGTGGCAC
MLH1 c.129dupA	AATATGTACATTAGAGTAGTTG	CAGAGAAAGGTCCTGACTC
MLH1 c.1831delA	CATTTGGATGCTCCGTTAAAGC	ACCCGGGTGGAAATTTTATTTG
BAT25 markers MSI	TACCAGGTGGCAAAGGGCA	TCTGCATTTTAACTATGGCTC-HEX
BAT 26 marker MSI	CTGCGGTAATCAAGTTTTTAG	AACCATTCAACATTTTTAACCC-HEX
